# Are global health commitments relevant in low-income countries? Considering discussions at Davos 2026

**DOI:** 10.1093/heapol/czag108

**Published:** 2026-08-31

**Authors:** Martina Mchenga

**Affiliations:** Independent Researcher, Lilongwe, Malawi

**Keywords:** health systems strengthening, low-income countries, health governance, health policy implementation, health reform, health systems resilience

## Abstract

At the 2026 World Economic Forum Annual Meeting in Davos, health was positioned as central to economic resilience, productivity, and geopolitical stability. Four themes dominated the global health agenda: artificial intelligence–enabled health systems, investible health system resilience, women’s health as an economic investment opportunity, and mental health as a macroeconomic priority. While these agendas reflect important global concerns, they are being advanced during a period characterized by shrinking development assistance, fiscal stress, debt pressures, and uneven institutional capacity across many low-income countries (LICs). This commentary argues that the central question is not only whether these agendas can be implemented in LICs but also whether they represent relevant and efficient priorities within resource-constrained health systems. Drawing on literature from health policy and systems research, health financing, implementation science, and political economy, the paper examines how current global health agendas frequently assume levels of data readiness, financing stability, administrative capability, workforce availability, and governance capacity that are not consistently present in constrained settings. On this basis, neither technological innovation nor investment mobilization can be treated as inherently valuable or as a substitute for contextualized priority setting. Proposed investments should be assessed against domestic disease burdens, societal preferences, system-wide effects, and feasible alternative uses of scarce resources. Durable progress in LICs will depend on strengthening countries’ capacity not only to implement reforms but also to determine which reforms warrant priority, allocate resources efficiently, and sustain foundational health system functions, including public financing, workforce development, information systems, regulatory capability, and accountable governance. The commentary concludes by calling for a context-sensitive and system-wide approach to global health agenda-setting.

Key messages

- Global health agendas increasingly emphasize innovation, technology, and investment mobilization, but often understate whether these priorities are relevant, efficient, or feasible in low-income country contexts.
- Artificial intelligence, resilience financing, women’s health investment, and mental health scale-up depend on underlying system capacity, including reliable data, sustainable financing, workforce capability, public stewardship, and both supply- and demand-side conditions.
- Under binding resource constraints, new investments carry opportunity costs and should be assessed against alternative interventions that may generate greater health and equity gains.
- Private and blended finance can complement, but not substitute for, public financing of essential health system functions that generate broad social benefits but limited financial returns.
- Future global health agendas should move beyond modular, predetermined solutions towards contextualized priority setting, domestic preferences, allocative efficiency, and system-wide assessment.

## Introduction

Health featured prominently at the 2026 World Economic Forum in Davos as central to economic resilience, productivity, and social stability (WHO 2026). Four priorities dominated discussions: artificial intelligence (AI)–enabled health systems (Farrugia 2026), investible health system resilience (Nataf 2026), women’s health as an underfunded investment area (Stroman and Bishen 2026), and mental health as a macroeconomic concern linked to productivity and social cohesion (Doustdar 2026). Although these priorities emerged from a high-level global policy forum rather than a formal priority-setting process for low-income countries (LICs), they are increasingly shaping international health discourse, investment narratives, and development partnerships. Their relevance and feasibility, however, are likely to vary across countries according to local health needs, fiscal constraints, and policy priorities.

These priorities are being advanced as many LICs face declining development assistance, rising debt burdens, limited domestic revenues, workforce shortages, and fragmented service delivery systems (Huckstep et al. 2025, OECD 2025). Such constraints raise questions not only about implementation but also about whether these investments represent the best use of scarce resources.

While each Davos priority addresses an important challenge, the agenda largely presents them as discrete innovations or investment opportunities. This risks promoting a modular approach to health system strengthening, in which individual interventions are advanced without sufficient attention to the system capacities needed to implement and sustain them (Adam and de Savigny 2012). Health policy and systems research instead emphasizes that the success of innovations depends on the strength and functioning of the systems into which they are introduced (Sheikh and Abimbola 2022).

This commentary argues that implementation feasibility has become a critical constraint in translating global health ambitions into sustainable gains in LICs. It focuses on four interrelated constraints, financing, workforce availability, institutional capability, and health system absorptive capacity (defined as the ability of health systems to adopt, integrate, and sustain new policies, technologies, and organizational innovations within existing institutional and operational constraints) (Godfrey et al. 2022), to examine how they shape the feasibility of the four Davos priorities.

## Artificial intelligence–enabled health systems

AI-enabled health systems featured prominently in Davos discussions, where they were promoted as tools to improve efficiency, support clinical decision-making, strengthen surveillance, and expand access to care (Farrugia 2026).

However, their successful adoption depends on reliable electricity, interoperable digital infrastructure, effective procurement and maintenance systems, appropriate data governance, and a sufficiently skilled workforce (WHO 2019; Nathan et al. 2025). In many LICs, these prerequisites remain weak.

The emphasis on AI also risks overlooking the quality of the data on which these systems depend. Routine health data are often incomplete, inconsistent, fragmented across parallel platforms, and underused for clinical, managerial, and resource-allocation decisions (Labrique et al. 2018). AI applied to poor-quality data may simply automate existing inaccuracies rather than improve decision-making or health system performance.

Moreover, investments in digital infrastructure, software, cybersecurity, maintenance, and specialized personnel carry opportunity costs and may divert resources from routine information systems, workforce development, essential medicines, and frontline services. Where AI initiatives rely on parallel reporting systems or donor-funded pilots, they may also increase fragmentation and widen existing inequalities.

AI should therefore be adopted only where data readiness, institutional capacity, and comparative value justify the investment.

## Investible resilience

Davos 2026 positioned health system resilience as an investible opportunity capable of attracting private capital, blended finance, and market-based financing mechanisms (Nataf 2026). This reflects broader efforts to frame resilience as both a health and economic security priority.

However, many LICs continue to face severe fiscal constraints, including rising debt burdens, narrow tax bases, inflationary pressures, and declining concessional financing (World Bank 2017, Mawejje 2025). Without sustainable domestic financing, resilience initiatives risk dependence on fragmented donor support or short-term project funding (Bayat et al. 2023, WHO and The World Bank 2025).

Greater reliance on private and blended financing also raises governance and equity concerns because private and public finance serve different objectives. Private investors typically require risk-adjusted financial returns, whereas public financing supports essential functions that generate broad social benefits without producing direct revenue. Investment-oriented approaches may therefore favour commercially viable activities while underinvesting in rural primary care, disease surveillance, community health systems, workforce development, and supply-chain capacity (Nabyonga-Orem et al. 2019). In settings with weak regulatory oversight, they may also increase financing fragmentation and weaken public stewardship (Montagu and Goodman 2016).

Private finance should complement, not replace, strong public financing, stewardship, and explicit priority setting to maximize health and equity gains within limited fiscal space (OECD 2023, Okyere et al. 2024).

## Women’s health

Women’s health emerged at Davos as a major underinvested area associated with substantial economic and productivity gains (Stroman and Bishen 2026).

However, translating investment commitments into health gains depends on delivery systems capable of providing accessible, affordable, and high-quality care (Kruk et al. 2018). Many LICs continue to face workforce shortages, weak referral systems, stockouts, user fees, geographic inequalities, and underperforming primary healthcare systems that limit access and continuity of care, particularly in rural and underserved areas (Alegre et al. 2024).

Framing women’s health primarily in terms of economic returns may also narrow policy priorities towards interventions with measurable productivity gains while overlooking broader gender, equity, and rights considerations (Morgan et al. 2016). Similarly, targeted investment programmes implemented outside broader primary healthcare systems risk further fragmenting service delivery (Siqueira et al. 2021).

Lasting improvements therefore depend on strengthening the primary healthcare systems that deliver women’s health services rather than relying on stand-alone investment programmes (Hanson et al. 2022).

## Mental health

Mental health emerged at Davos as a public health and economic priority linked to productivity and social stability (Doustdar 2026). However, designating mental health as a global priority does not by itself overcome the supply- and demand-side barriers that constrain access in many LICs. Services frequently face inadequate financing, shortages of trained personnel, weak referral and community-support systems, and limited integration into primary care, and concentration in urban tertiary facilities, restricting access for rural and underserved populations (WHO 2024; Acuña-Rodríguez et al. 2025).

Demand is further constrained by stigma, limited mental health literacy, culturally specific understandings of distress, concerns about confidentiality, low trust in services, and the direct and indirect costs of seeking care (Sarikhani et al. 2021). These barriers influence whether individuals recognize a need for care, seek support, and remain engaged with treatment, limiting effective coverage even where services are available (van den Broek et al. 2023).

Sustainable scale-up, therefore, requires context-specific, long-term investment in workforce training, supervision, referral pathways, continuity of care, community engagement, and interventions that reduce stigma and strengthen trust in services (WHO 2022).

## Towards a context-sensitive and system-wide global health agenda

The central challenge across contemporary global health agendas is not only whether proposed reforms can be implemented, but whether they reflect the most relevant and efficient priorities for resource-constrained health systems (WHO 2016). Innovations promoted through global policy forums should not be assumed to align automatically with domestic disease burdens, institutional capacity, or public preferences.

A stronger approach begins with contextualized priority setting. Countries should assess competing health needs using local evidence, societal preferences, and explicit consideration of opportunity costs (WHO 2016). Under binding resource constraints, investments should be judged not only by feasibility but also by whether they generate greater health and equity gains than available alternatives.

This also requires moving beyond a modular approach that evaluates individual innovations in isolation (Adam and de Savigny 2012, Swanson et al 2012). Proposed investments should be assessed for how they interact with financing, workforce capacity, service delivery, governance, information systems, and existing inequalities and whether they strengthen or weaken these core health system functions.

## Conclusion

The Davos 2026 agenda reflects growing recognition of health as central to economic resilience and social stability. However, global priorities should not be assumed to be universally relevant or efficient across LICs. Future agenda-setting should be grounded in contextualized priority setting, explicit consideration of opportunity costs, and system-wide assessment to ensure that innovations strengthen health systems while maximizing health and equity gains.

## Data Availability

No new data were generated or analysed in support of this commentary. All material discussed is available in the published sources cited in the reference list.

## Reflexivity statement

The author is a health economist and global public health researcher based in sub-Saharan Africa, with professional experience spanning academia, health financing policy, and health systems strengthening across multiple African countries. The author has worked with ministries of health, international organizations, and research institutions on issues related to health financing, universal health coverage, implementation of health reforms, and policy translation. These experiences informed the interpretation of contemporary global health agendas and implementation challenges discussed in this commentary, particularly within resource-constrained settings.
